# A mitochondrial therapeutic reverses visual decline in mouse models of diabetes

**DOI:** 10.1242/dmm.020248

**Published:** 2015-07-01

**Authors:** Nazia M. Alam, William C. Mills, Aimee A. Wong, Robert M. Douglas, Hazel H. Szeto, Glen T. Prusky

**Affiliations:** 1 Department of Physiology and Biophysics, Weill Cornell Medical College, New York, NY, USA; 2 Burke Medical Research Institute, White Plains, NY, USA; 3 Research Program in Mitochondrial Therapeutics, Department of Pharmacology, Weill Cornell Medical College, New York, NY, USA; 4 Department of Ophthalmology and Visual Sciences, University of British Columbia, Vancouver, British Columbia, Canada

**Keywords:** Diabetic retinopathy, RPE, SS-31, MTP-131, Bendavia, Insulin resistance, Hyperglycemia, Optomotor, Spatial vision, Cardiolipin, OKT, Mouse

## Abstract

Diabetic retinopathy is characterized by progressive vision loss and the advancement of retinal micoraneurysms, edema and angiogenesis. Unfortunately, managing glycemia or targeting vascular complications with anti-vascular endothelial growth factor agents has shown only limited efficacy in treating the deterioration of vision in diabetic retinopathy. In light of growing evidence that mitochondrial dysfunction is an independent pathophysiology of diabetes and diabetic retinopathy, we investigated whether selectively targeting and improving mitochondrial dysfunction is a viable treatment for visual decline in diabetes. Measures of spatial visual behavior, blood glucose, bodyweight and optical clarity were made in mouse models of diabetes. Treatment groups were administered MTP-131, a water-soluble tetrapeptide that selectively targets mitochondrial cardiolipin and promotes efficient electron transfer, either systemically or in eye drops. Progressive visual decline emerged in untreated animals before the overt symptoms of metabolic and ophthalmic abnormalities were manifest, but with time, visual dysfunction was accompanied by compromised glucose clearance, and elevated blood glucose and bodyweight. MTP-131 treatment reversed the visual decline without improving glycemic control or reducing bodyweight. These data provide evidence that visuomotor decline is an early complication of diabetes. They also indicate that selectively treating mitochondrial dysfunction with MTP-131 has the potential to remediate the visual dysfunction and to complement existing treatments for diabetic retinopathy.

## Abstract (precis)

**Summary:** Visual decline in mouse models of diabetes is reversed, independently of treating other disease symptoms, by treatment with MTP-131, a water-soluble peptide that selectively targets cardiolipin and improves mitochondrial bioenergetics.

## INTRODUCTION

Diabetic retinopathy is a leading cause of progressive vision loss and blindness. It is characterized by occlusion and leakage of retinal vessels, which leads to macular edema in its non-proliferative phase, and angiogenesis and retinal detachment in its proliferative phase. Whereas vascular endothelial growth factor expression is necessary for the angiogenesis (Stefanini et al., 2014), treatment with anti-vascular endothelial growth factor agents is able to improve visual function in <30% of patients. Likewise, therapies aimed at managing the symptoms of metabolic dysfunction have shown limited efficacy in slowing the progression of diabetic retinopathy; diabetic complications develop in ∼20% of patients under strict glycemic or blood-pressure control (Antonetti et al., 2012)*.* Thus, earlier detection of risk for diabetes and diabetic retinopathy, and intervention with novel therapeutics before irreversible retinal damage occurs, have great potential to improve treatment.

There is growing recognition that retinal dysfunction (Barber et al., 2005; Ly et al., 2011; Martin et al., 2004; Murakami and Yoshimura, 2013; van Dijk et al., 2009) and impaired visual behavior is present in human diabetics and in animal models before retinal vascular changes are evident (Akimov and Rentería, 2012; Aung et al., 2013; Hardy et al., 1992; Jackson et al., 2012; Kirwin et al., 2011). Thus, the identification of visual dysfunction early in the course of diabetes may provide an advanced opportunity for therapeutic intervention. A promising candidate for early intervention in diabetic retinopathy is the remediation of mitochondrial dysfunction. Diabetic complications in the non-proliferative phase of diabetic retinopathy are associated with metabolic pathways that are upregulated by sustained hyperglycemia: increased polyol pathway flux; increased formation of advanced glycation end products and their receptors; activation of protein kinase C; and increased hexosamine pathway flux (Forbes and Cooper, 2013). It has been proposed that the pathways are linked by the mitochondrial production of reactive oxygen species resulting from increased metabolic flux through the electron transport chain. This is supported by evidence that normalizing mitochondrial superoxide production can mitigate hyperglycemic damage (Brownlee, 2001; Nishikawa et al., 2000).

Indeed, mitochondria are simultaneously a major source of intracellular reactive oxygen species and the target of oxidative damage, and evidence of mitochondrial oxidative stress is present when histopathological abnormalities arise in diabetic retinopathy (Du et al., 2003; Kowluru and Zhong, 2011). Altered mitochondrial structure, including swelling and loss of cristae, the accumulation of defects in mitochondrial DNA, and a reduction of transport proteins (Kowluru and Zhong, 2011; Madsen-Bouterse et al., 2010b; Santos and Kowluru, 2013), which may be independent of hyperglycemia (Zhong and Kowluru, 2011), have also been reported. In addition, impairment of retinal pigment epithelium mitochondria is associated with increased oxidative stress, reduced ATP, and compromised autophagic and phagocytic capacities (He et al., 2010; Kanwar et al., 2007; Li et al., 2014; Madsen-Bouterse et al., 2010a). Taken together, these data indicate that apoptotic stress in the retina has the capacity to induce the hallmark microvascular injury of diabetic retinopathy, and that improving mitochondrial function may be an effective treatment.

Whereas antioxidants have shown promise in preclinical studies as a therapy for diabetic retinopathy (Huang et al., 2013; Nawaz et al., 2013; Xie et al., 2008), they have not been effective in clinical trials, possibly owing to their inability to penetrate mitochondria. This problem can be overcome with the use of MTP-131 (also known as SS-31), a water-soluble mitochondria-targeting peptide that attenuates mitochondrial reactive oxygen species production and cytochrome c release (Huang et al., 2013; Li et al., 2011; Szeto and Birk, 2014; Zhao et al., 2004). We thus tested the hypothesis here that MTP-131 can remediate visual impairment in mouse models of diabetes.

**TRANSLATIONAL IMPACT**

**Clinical issue**

Diabetes is a chronic condition characterized by high blood sugar levels (hyperglycemia). Insulin usually controls blood glucose, but in individuals with diabetes either insufficient insulin is made (type-1 diabetes) or the cells that normally respond to insulin become insulin resistant (type-2 diabetes). A common long-term complication of diabetes is diabetic retinopathy, a condition that can cause blindness, in which blood vessels in the back of the eye narrow, leak and proliferate abnormally. Current treatments for visual dysfunction in individuals with diabetes aim: to manage hyperglycemia, obesity and retinal swelling in the early, non-proliferative phase of diabetic retinopathy; to improve the clarity of the optical axis; or to control retinal angiogenesis (new blood vessel formation) and its consequences in the proliferative stage. These treatments have limited efficacy, and only slow, but do not reverse, visual decline.

**Results**

Mitochondrial dysfunction is thought to be involved in the pathogenesis of diabetic retinopathy; here, the authors describe a novel approach to treating diabetic visual dysfunction based on targeting and treating mitochondrial dysfunction with a water-soluble peptide (MTP-131). The authors report that a progressive decline in spatial visuomotor function (movements that are dependent on sight) emerges early in mouse models of type-1 and type-2 diabetes, before the typical symptoms of diabetes, such as hyperglycemia and obesity, develop. Both systemic and eye-drop application of MTP-131 early in the course of visual decline prevented further decline and fully reversed visual dysfunction without normalizing aberrant glucose clearance or hyperglycemia and weight. Equivalent treatment later in disease, after much more severe visual dysfunction was manifest, also substantially restored function.

**Implications and future directions**

These data indicate that mitochondrial dysfunction, which can be detected with spatial measures of visuomotor function, is an early and treatable pathophysiology of diabetes that is independent of the other symptoms of metabolic dysfunction. They also indicate that improving mitochondrial function can treat the visual dysfunction without remediating glucose dysregulation or obesity. Thus, mitochondrial-based treatment on its own, or in combination with current therapies, might provide a new, more-effective approach to the treatment of the visual consequences of diabetes at any stage of the disease. Since MTP-131 has shown an exceptional human safety profile in clinical trials for other conditions that involve mitochondrial dysfunction, probably because of its lack of effect on normal mitochondria, it has the potential to be rapidly translated into clinical trials for the treatment of diabetic retinopathy.

## RESULTS

### Characterization of diabetic mouse models

Type-1 diabetes is an autoimmune disease that leads to loss of insulin-producing β-cells in the pancreas, which is often modeled in animal studies with injections of streptozotocin (STZ). We administered STZ to C57BL/6 mice fed a normal diet (ND) for 5 days at 8 weeks of age (ND+STZ). Type-2 diabetes is characterized by insulin resistance and the inability of β-cells to upregulate their insulin production. For modeling this, mice were fed a diabetic diet (DD) high in fat and carbohydrates from 4 weeks of age, and a third group received both DD and STZ (DD+STZ). Control mice were fed a ND throughout the study.

**Fig. 1.**
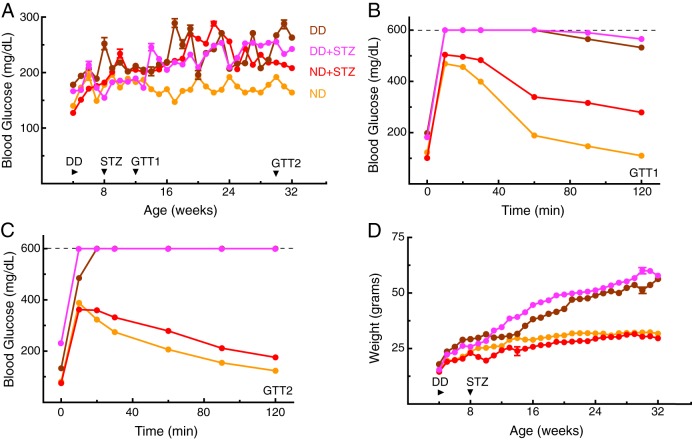
**Metabolic dysfunction in mouse models of diabetes.** (A) Elevated blood glucose emerged in ND+STZ, DD and DD+STZ groups by 15 weeks of age [DD=215±4.35 mg/dl (*n*=7); DD+STZ=235±2.28 mg/dl (*n*=6); ND+STZ=205±3.14 mg/dl (*n*=12), significantly higher than ND=161±1.73 mg/dl (*n*=7), *P*<0.01] and was sustained until 32 weeks [DD=263±1.35 mg/dl (*n*=4); DD+STZ=253±2.22 mg/dl (*n*=3); ND+STZ=208±3.64 mg/dl (*n*=9), significantly higher than ND=164±2.73 mg/dl (*n*=5), *P*<0.01]. (B,C) Evidence of impaired glucose clearance on a glucose tolerance test was present in each diabetic model when measured at 12 (B; GTT1) and 30 (C; GTT2) weeks, with DD groups being the most impaired. Dashed lines indicate the upper limit of glucometer sensitivity. (D) Abnormal weight gain emerged in the diabetic models by 15 weeks (DD=35.2±3.11 g, DD+STZ=41.3±1.42 g, ND+STZ=24.9±2.31 g), which was significantly different from ND (29.0±2.14 g), *P*<0.05. Data are mean±s.e.m. (error bars are often occluded by the data symbols).

Elevated resting blood glucose (non-fasted) was present in each diabetic model by 15 weeks, which was sustained at 32 weeks (Fig. 1A). Evidence of insulin resistance was also present in the results of a glucose tolerance test administered at 12 (Fig. 1B) and 30 weeks (Fig. 1C), with DD groups showing the greatest impairment. Elevated bodyweight emerged by 15 weeks in groups fed a DD, and by 32 weeks, mice in the DD groups were two-fold heavier than mice fed a ND (Fig. 1D, ∼56 g versus ∼29 g).

### Progressive decline of visual function in diabetic mouse models

**Fig. 2.**
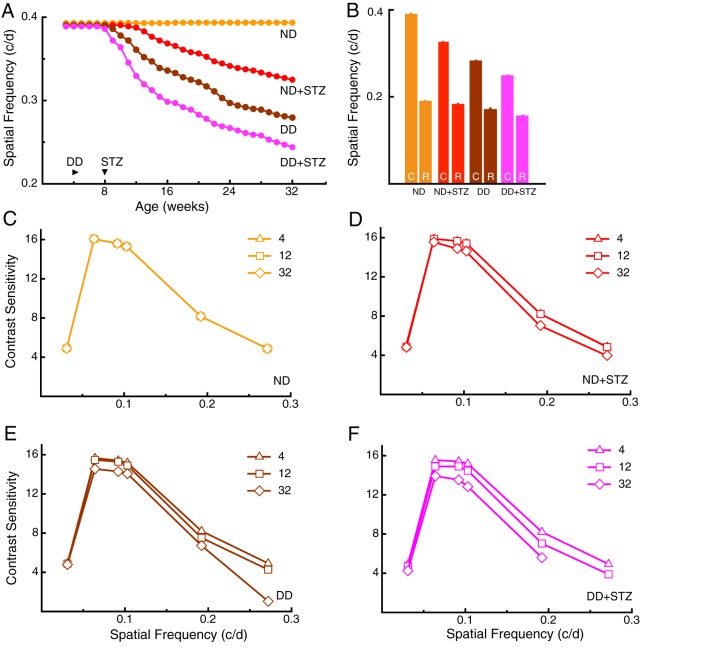
**Progressive decline of visuomotor function in diabetic models.** (A) SF thresholds under photopic (cone-mediated) conditions did not change in ND mice from 12 [0.393±0.006 c/d (*n*=7)] to 32 weeks [0.394±0.001 c/d (*n*=5), *P*>0.05]. Reduced thresholds (average of both eyes) emerged in ND+STZ mice at 12 weeks [0.387±0.003 c/d (*n*=12), *P*<0.05], with a 17% decline by 32 weeks [0.325±0.001 c/d (*n*=9), *P*<0.01]. Reduced thresholds emerged in DD mice by 9 weeks [0.386±0.002 c/d (*n*=7), *P*<0.01], with a 29% decline by 32 weeks [0.280±0.001 c/d (*n*=4), *P*<0.01]. A reduction emerged in DD+STZ mice at 8 weeks [0.327±0.001 c/d (*n*=6), *P*<0.01], with a 38% decline by 32 weeks [0.244±0.001 c/d (*n*=3), *P*<0.01]. (B) Cone-mediated function was more compromised than rod function in diabetic models at 28 weeks. As previously reported (Altimus et al., 2010), rod thresholds (photopic; R) are significantly lower (0.19225±0.000324 c/d) than cone (C) thresholds (0.3935±0.0006 c/d, *P*<0.01, *n*=4) in ND mice. Both rod and cone thresholds were reduced from normal in all diabetic models, with the most impairment in cone-mediated function: ND+STZ (cone versus rod: 0.331±0.0005 c/d versus 0.18624±0.0000342 c/d, *P*<0.01); DD (cone versus rod: 0.286±0.00634 c/d versus 0.17268±0.000325 c/d, *P*<0.01); DD+STZ (cone versus rod: 0.253±0.0000233 c/d versus 0.15813±0.000435 c/d, *P*<0.01). (C-F) Progressive decline of CS in diabetic models. (C) Normal CS was present in ND mice when measured at 4, 12 and 32 weeks. (D) CS in ND+STZ mice decreased moderately between 12 and 32 weeks at SFs above 0.06 c/d. (E) Decreased CS was evident at 12 weeks in DD mice and progressed by 32 weeks, particularly at the highest SF tested. (F) DD+STZ mice displayed the most dysfunction over time, such that by 32 weeks, they did not respond at the highest SF tested.

Spatial frequency (SF) and contrast thresholds for optokinetic tracking were measured through each eye once/week from 3-32 weeks, using a virtual optokinetic system (Prusky et al., 2004). SF thresholds near 0.39 cycles/degree (c/d) were measured in ND mice (Fig. 2A) up to 32 weeks; performance was comparable to previously published values (Prusky et al., 2004, 2006). Loss of function emerged by 12 weeks in ND+STZ mice, which, by 32 weeks, was reduced by 17%. Visual dysfunction emerged earlier (at 9 weeks) and declined more (29%) in DD mice. In DD+STZ mice, visual dysfunction emerged the earliest (at 8 weeks), and declined the most (38%), by 32 weeks.

We have previously defined the luminance conditions in the virtual optokinetic system that are able isolate cone- and rod-only function (Alam et al., 2015; Altimus et al., 2010), and as such, the lighting conditions used to record the thresholds in Fig. 2A were selective for cone-mediated function. In order to measure rod-mediated function in the same animals, we configured the virtual optokinetic system to record rod-driven thresholds in low light conditions. Fig. 2B shows a comparison of cone- and rod-selective function at 28 weeks. Whereas cone function was reduced by 16% in ND+STZ mice, 27% in DD mice and 36% in DD+STZ mice, rod function was proportionally less affected; ND+STZ=3%; DD=10%, and DD+STZ=18%. Thus, the loss of visual function in the diabetic models was primarily due to compromised cone-mediated responses.

Measures of CS also revealed evidence of visual decline that was graded by group. Adult-like CS (Douglas et al., 2005; Prusky et al., 2004) was present in ND mice at 4, 12 and 32 weeks (Fig. 2C). CS in ND+STZ mice (Fig. 2D) was normal at 4 and 12 weeks, but was slightly reduced by 32 weeks at SFs above 0.06 c/d. CS in DD mice was marginally lower at high SFs between 4 and 12 weeks, and by 32 weeks, was reduced at all SFs above 0.03 c/d (Fig. 2E). DD+STZ mice showed loss of function from 4 to 12 weeks comparable with DD mice, but showed a greater decline by 32 weeks (Fig. 2F).

### MTP-131 treatment reversed visual decline in diabetic mouse models

**Fig. 3.**
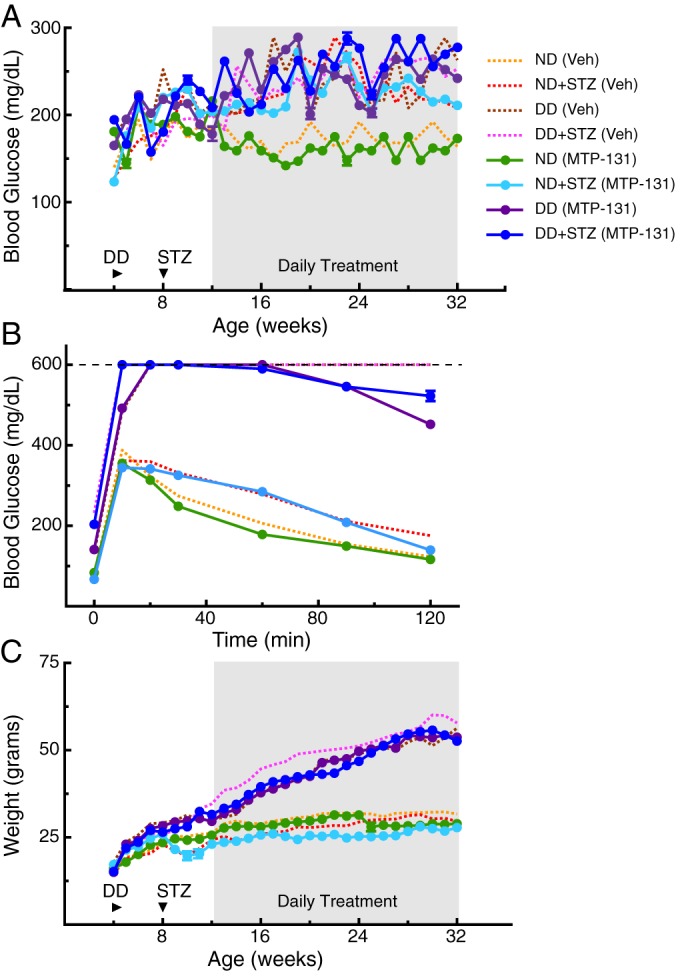
**Metabolic dysfunction was not improved by daily systemic treatment with MTP-131.** Daily MTP-131 treatment is indicated by shading. Dotted lines are traces from Fig. 1, depicting the effect of Veh treatment. (A) MTP-131 treatment did not reduce resting fed blood glucose in any of the experimental groups. (B) MTP-131 had no effect on glucose clearance at 32 weeks in any experimental group. (C) MTP-131 did not mitigate weight gain in the groups fed a DD.

**Fig. 4.**
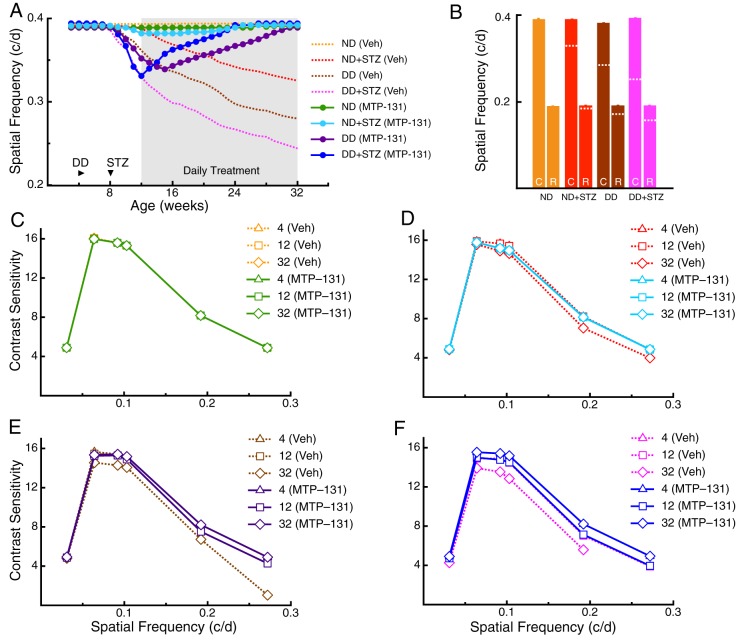
**Reversal of visual dysfunction following daily systemic treatment with MTP-131.** Daily MTP-131 treatment is indicated by shading. Dotted lines are traces from Fig. 1, depicting the effects of Veh treatment. (A) Improvement of SF function was present within 2 weeks of treatment in ND+STZ mice [MTP-131 versus Veh; 0.382±0.001 c/d (*n*=7) versus 0.377 c/d±0.006 (*n*=12)], within 3 weeks in DD mice [MTP-131 versus Veh: 0.342 c/d±0.001 (*n*=8) versus 0.347 c/d±0.003 (*n*=7)] and within 1 week in DD+STZ mice [MTP-131 versus Veh: 0.340±0.001 c/d (*n*=11) versus 0.313±0.002 c/d (*n*=6)]. Full restoration of function was achieved by 24 weeks in ND+STZ (*n*=4) and DD+STZ (*n*=8) mice, and by 31 weeks in DD mice (*n*=5). ND (MTP-131) did not differ statistically from ND (Veh) at any age (*n*=6, *P*>0.05). (B) Cone- and rod-mediated function was restored by MTP-131 treatment, as measured at 28 weeks of age (*P*>0.05); dotted lines depict the results of Veh treatment abstracted from Fig. 1. (C-F) MTP-131 restored normal CS in each experimental group by 32 weeks.

Treatment groups were injected with MTP-131 (1 mg/kg) [vehicle (Veh), 0.9% saline], subcutaneously once/day from 12 weeks, an age by which evidence of visuomotor dysfunction had developed in each of the diabetic models (i.e. Fig. 2). The treatment did not alter the level of non-fasted resting blood glucose (Fig. 3A), and did not change the rate of glucose clearance measured at 30 weeks (compared with the Veh group; Fig. 3B). Likewise, the treatment did not correct the elevated bodyweight of the DD groups (Fig. 3C). Improvement of visual function, however, was present in each of the diabetic groups. Fig. 4A shows that improvement of the SF threshold for opto-kinetic tracking was present in DD+STZ mice 1 week after treatment, within 4 weeks in DD mice and within 6 weeks in ND+STZ mice. Improvement continued thereafter in all groups until thresholds were restored to normal values by 24 weeks in DD+STZ mice, by 25 weeks in ND+STZ mice and by 31 weeks in DD mice. MTP-131 treatment did not alter function in the control (ND) group. Cone-and-rod selective dysfunction in the MTP-131-treated mice were both fully reversed in ND+STZ and DD+STZ groups, and substantially remediated in the DD group (Fig. 4B; compare with 28 weeks in Fig. 2B). MTP-131 treatment improved CS in a similar manner; MTP-131 did not affect CS in ND mice (Fig. 4C), but it restored thresholds to near-normal values in ND+STZ (Fig. 4D), in DD (Fig. 4E) and in DD+STZ mice (Fig. 4F). Ophthalmic examinations of mice presented in Figs. 3 and 4 revealed only sporadic ocular abnormalities, none of which were related to group or treatment. All animals were assessed at 12 weeks of age, prior to starting any treatment, and at 32 weeks, prior to euthanasia, using a modification of the MacDonald-Shadduck Scoring System described elsewhere (Altmann et al., 2010). Ophthalmic examinations identified three animals with abnormal visual thresholds at 12 weeks, in the form of dense corneal infiltrates or central opacities, likely due to grooming or fighting. The mice were removed from the study. Two left eyes and two right eyes showed small punctate corneal opacities (less than 25% corneal involvement score=1), either in the inferior or nasal region of four animals prior to 12 weeks of age, but that animals remained in the study and were randomly assigned into groups. Ophthalmic examinations were performed again at 32 weeks of age, and no animals were identified with ocular problems.

### Efficacy of MTP-131 applied via eye drops

**Fig. 5.**
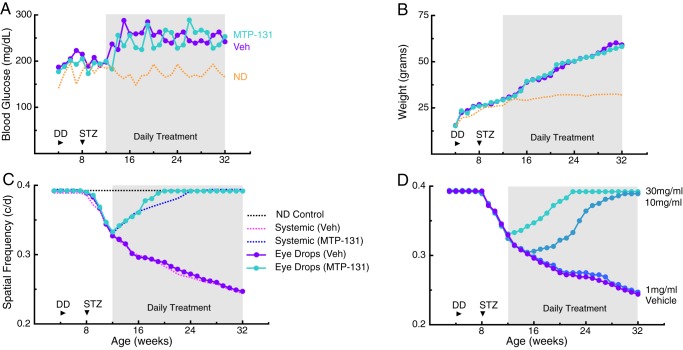
**Daily administration of MTP-131 in eye drops from 12 weeks reversed visual decline.** (A,B) 30 mg/ml MTP-131 did not affect resting blood glucose (A; MTP-131 versus Veh, *P*>0.05 versus *P*<0.01 for two-way ANOVA on age, interaction and treatment condition compared with ND group after 12 weeks) or body weight (B; MTP-131 versus Veh, *P*>0.05 versus a *P*<0.001 for two-way ANOVA on age, interaction and treatment condition compared with ND group). Dotted lines represent control data from ND mice in Fig. 1. (C) SF function was restored to control values by 20 weeks, 4 weeks earlier than with systemic treatment [*P*>0.05, eye drops (Veh) versus systemic (Veh); *P*<0.001 for eye drops (MTP-131) versus ND from 7-19 weeks of age, *P*<0.05 from 20 weeks of age; eye drops (MTP-131) versus systemic (MTP-131), *P*<0.01 between 17 and 23 weeks of age (Fig. 2A)]. (D) Dose-dependent effect of MTP-131 eye drop treatment; 1 mg/ml had no effect (*P*<0.01 for 30 mg/ml group from 13 weeks of age compared with Veh and 1 mg/ml groups; *P*<0.01 for 10 mg/ml group from 16 weeks of age compared with Veh and 1 mg/ml groups; *P*<0.001 for 30 mg/ml group versus 10 mg/ml group from 13 to 29 weeks; *P*>0.05 for 1 mg/ml group versus Veh at all points).

We hypothesized that the benefit of systemic MTP-131 treatment was related to its action on the retina, as MTP-131 did not correct weight gain or high blood glucose. In an effort to deliver MTP-131 to the eye, we investigated whether eye drop application of the peptide would also treat diabetic visual dysfunction. Fig. 5A-C shows that resting blood glucose and body weight measures were unaffected by MTP-131 or placebo (Veh) eye drop application. In Fig. 5C, Veh-treated mice exhibited a SF threshold decline comparable with that produced by systemic Veh treatment (Fig. 2A, magenta line). However, the decline in the MTP-131 eye drop-treated group was reversed after 1 week of treatment, and normal function was reinstated by 20 weeks – 4 weeks earlier than with systemic MTP-131 treatment in the same model.

In a separate cohort of mice, we investigated whether the timing and rate of visual recovery following the application of MTP-131 was dose dependent (Fig. 5D). Improved function was observed within 1 week of treatment with 30 mg/ml, and within 5 weeks with 10 mg/ml. Treatment with 1 mg/ml did not lead to improvement. As with systemic MTP-131 treatment, the restoration of function with eye drop application of MTP-131 was independent of overt changes in the quality of the optical axis (data not shown).

### MTP-131 reversed more severe visual dysfunction

**Fig. 6.**
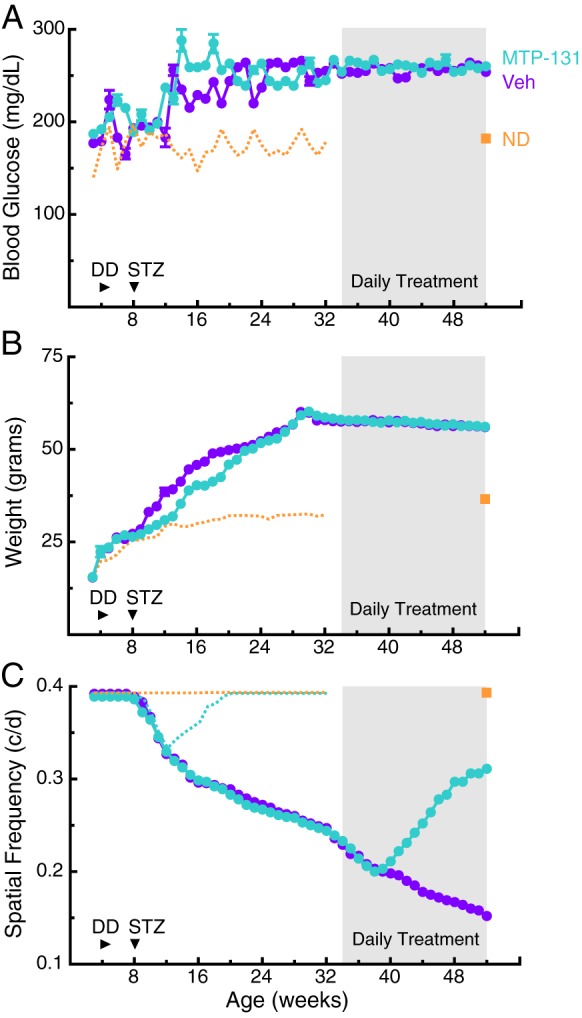
**Daily administration of MTP-131 in eye drops reversed the severe visual dysfunction present later in the course of the disease.** (A,B) Treatment with MTP-131 in eye drops (30 mg/ml) from 34 weeks (shading) in DD+STZ mice did not change markers of metabolic dysfunction [A; resting blood glucose at 52 weeks MTP-131 treatment versus Veh: 260 mg/dl±2.64 (*n*=8) versus 254±2.11 mg/dl (*n*=7), *P*=0.1363; compared with ND: 173±3.06 mg/dl (*n*=4)] or of body weight (B; at 52 weeks MTP-131 treatment versus Veh: 56.1±2.5 g versus 56.0±2.3 g, *P*=0.1021; compared with ND: 36.5±3.1 g). Orange dotted lines represent traces from control ND mice in Fig. 1. Orange squares are measures in age-matched control mice at 52 weeks. (C) Reversal of visual decline was evident after 6 weeks of drug treatment (MTP-131 versus Veh: 0.211 c/d±0.003 versus 0.198±0.003 c/d, *P*<0.05); recovery to 80% normal was evident by 52 weeks (MTP-131 versus Veh: 0.311±0.003 c/d versus 0.152±0.003 c/d; compared with ND: 0.393±0.00 c/d1). Dotted orange line depicts the function of ND mice; teal line is the effect of MTP-131 eye drop treatment (taken from Fig. 5C).

To determine whether MTP-131 application was able to remediate more pronounced visual dysfunction than that at 12 weeks (i.e. Fig. 5), eye drop treatment with MTP-131 (30 mg/ml) was initiated in a group of DD+STZ mice at 34 weeks, an age at which the SF threshold in untreated animals was reduced by ∼50% (Fig. 6C). As in previous experiments, MTP-131 treatment had no effect on resting blood glucose (Fig. 6A) or body weight (Fig. 6B). Unlike the effect on visual function with treatment from 12 weeks, which showed a beneficial effect within 1 week (Fig. 5C), visual function continued to decline for 4 weeks after MTP-131 treatment in the group. At 5 weeks, however, visual decline was halted, followed by recovery at a rate similar to that with treatment from 12 weeks, reaching ∼75% of normal by 52 weeks (Fig. 6C). As stipulated by our animal protocol, the study was terminated at 52 weeks; thus, it is not known whether continued treatment would have led to more improvement. However, the rate of recovery appeared to slow after 48 weeks, likely indicating that complete recovery would not be achieved with more time. Whereas CS was measured in all of the eye drop studies, it changed in concert with measures of SF thresholds; thus, the data are not presented here.

## DISCUSSION

This study, which unfolded over the course of 2 years and used over 150 mice, is the most comprehensive examination to date of visual decline, and restoration with treatment, in rodent models of diabetes. It establishes a time course of the relationship between the emergence of metabolic dysfunction and visual decline in three diabetic models in the same mouse strain, it illuminates a role for mitochondrial dysfunction in the pathogenesis of diabetic visual complications and it demonstrates a clinically feasible approach to treating it.

We used a repeated low-dose STZ protocol to model type-1 diabetes, rather than the typical single high dose STZ protocol, to reduce the off-target toxic effects of STZ (Hayashi et al., 2006; Like and Rossini, 1976). This resulted in only a moderate increase in fed blood glucose (Hayashi et al., 2006; Leiter, 1982) and slightly abnormal glucose clearance, which may better model type-1 diabetes. Feeding a diet high in fat and carbohydrates was used to model type-2 diabetes, which led to an increase in body weight, abnormal glucose clearance and mild hyperglycemia. Although we did not measure insulin levels in this study, an increase in plasma insulin has previously been reported in diet-induced models of diabetes (Park et al., 2005; Surwit et al., 1988). For example, a decrease in insulin-stimulated glucose uptake in the heart has been reported within 1.5 weeks of initiating a high-fat diet in young C57BL/6 mice, which was reduced by ∼90% after 20 weeks (Park et al., 2005). In addition, plasma insulin was elevated within 2 weeks in young mice fed a high-fat diet, and remained elevated for up to 52 weeks (Park et al., 2005; Surwit et al., 1988; Winzell and Ahrén, 2004). Thus, the DD model enabled us to study the effect of insulin resistance on visual function. Indeed, the elevated blood glucose in DD mice most likely resulted from reduced glucose uptake by skeletal muscles and adipose tissue, due to loss of insulin signaling (Winzell and Ahrén, 2004). A group that combined DD with STZ treatment provided a model of advanced type-2 diabetes, which is characterized by defects in insulin secretion and insulin resistance.

A decline in visuomotor function was detected in each experimental model before changes in resting blood glucose emerged: a common clinical indicator of susceptibility to diabetes. The onset of visual decline was most rapid in the DD+STZ group and slowest in the ND+STZ group, suggesting that insulin resistance may have an early negative effect on visual function. Insulin receptors are expressed on both vascular and neural cells of the retina (Haskell et al., 1984), and are able to autophosphorylate and activate downstream kinases, as in other insulin-sensitive tissues (Reiter et al., 2003). Rats administered STZ have shown reduced insulin receptor signaling in the retina, especially in retinal endothelial cells, which may contribute to the early progression of diabetic retinopathy (Aizu et al., 2002; Fort et al., 2011; Kondo and Kahn, 2004; Reiter et al., 2003).

The study was designed such that daily MTP-131 treatment began 4 weeks after STZ treatment or 8 weeks after the initiation of a DD. As visual decline only began 4 weeks after STZ, MTP-131 treatment appeared to both reverse further decline, and prevent sustained visual dysfunction. MTP-131 treatment was also able to reverse more pronounced visual dysfunction in the DD group, but the time to full recovery was shortened. Unexpectedly, even though the DD+STZ mice had the most dramatic rate of visual decline, they had the most rapid response to MTP-131 treatment, showing improvement within 1 week of MTP-131 administration and full recovery after 12 weeks. The lack of effect of MTP-131 on blood glucose and body weight is in keeping with previous reports in STZ-administered rats (Huang et al., 2013). The more rapid onset of visual decline and the slower treatment response in the DD group are also consistent with insulin resistance as a cause of diabetic visual dysfunction. Because the addition of STZ has been shown to reduce diet-induced insulin resistance in C57BL/6 mice (Ning et al., 2011), this may account for the more rapid MTP-131 treatment effect in the DD+STZ group.

MTP-131 is a mitochondria-targeting peptide known to protect the structure of cristae and prevent mitochondrial swelling under ischemic conditions in numerous cell types (Birk et al., 2013; Liu et al., 2014; Szeto et al., 2011). By maintaining cristae membranes, MTP-131-treated tissues may be better able to sustain ATP synthesis and preserve vital ATP-dependent processes (Birk et al., 2013; Szeto et al., 2011). Although MTP-131 was known at the outset to selectively partition to the inner mitochondria membrane (Zhao et al., 2005), the mechanism by which MTP-131 protects mitochondrial cristae remained unclear until recently. It is now known, however, that MTP-131 has a high affinity for cardiolipin, a unique anionic phospholipid (Birk et al., 2013; Szeto, 2014). The conical shape of cardiolipin is required to maintain the curvature of cristae, and cardiolipin deficiency results in a loss of cristae and reduced mitochondrial respiration (Schlame and Ren, 2006). As cardiolipin contains four unsaturated acyl chains, it is readily peroxidized under oxidative conditions, which is consistent with reports that increased cardiolipin peroxidation and cardiolipin depletion are present in diabetic hearts (Ferreira et al., 2013; He and Han, 2014). High glucose has also been postulated to increase mitochondrial reactive oxygen species (Brownlee, 2001); thus, cardiolipin peroxidation could have the effect of reducing mitochondrial respiration. Interestingly, retinal mitochondria from diabetic mice show elevated superoxide levels and reduced glutathione, indicative of mitochondrial oxidative stress (Kanwar et al., 2007). In addition to a high reactive oxygen species environment, cardiolipin peroxidation is catalyzed by cytochrome c that is tightly bound to cardiolipin, and this cyt c/cardiolipin complex converts cytochrome c from an electron carrier to a peroxidase that can peroxidize cardiolipin (Kagan et al., 2005; Sinibaldi et al., 2010; Snider et al., 2013). MTP-131 appears to protect the architecture of mitochondrial cristae by reducing mitochondrial oxidative stress and preventing cytochrome c peroxidase activity (Birk et al., 2013; Zhao et al., 2004).

Mitochondrial abnormalities have been documented in insulin-resistant and diabetic states in human and animal studies, and it has been proposed that mitochondrial dysfunction may be the primary defect in obesity-related insulin resistance (Patti and Corvera, 2010). The causal mechanism underlying the mitochondrial dysfunction, however, is not fully understood. Although it is often assumed that hyperglycemia causes mitochondrial oxidative stress in diabetes, a recent study reported lower mitochondrial superoxide and mitochondrial respiratory activity in the kidneys of diabetic mice after STZ treatment (Dugan et al., 2013); this suggests a loss of functional mitochondria in the latter stages of diabetes (Sharma, 2015). MTP-131 could thus provide a novel approach for treating diabetes, based not on mitigating elevated blood glucose, but on enhancing mitochondrial bioenergetics through the preservation of mitochondrial cristae and improved efficiency of the electron transfer chain. MTP-131 has displayed an exceptional safety profile in Phase 1 clinical trials, likely due to its lack of effect on the function of normal mitochondria (Siegel et al., 2013). As the beneficial effects of MTP-131 appear to be independent of reducing circulating glucose, the treatment may also be useful in complementing therapies aimed at managing the typical symptoms of diabetes. Indeed, based on the results reported here, a clinical formulation of topical ophthalmic MTP-131 (Ocuvia) has entered a clinical trial for treating diabetic macular edema.

## MATERIALS AND METHODS

### Animal subjects

Experimental procedures on animals were conducted in accordance with the policies of the Weill Cornell Medical College Institutional Animal Care and Use Committee. One-hundred and fifty-one male C57BL/6 mice obtained from Charles River Laboratories at 3 weeks of age were group housed at the Burke Medical Research Institute vivarium. They were maintained at 68°-76°F with 30-70% relative humidity, and a photoperiod of 12 h light (06:00 lights on)/12 h dark (18:00 lights off).

### Disease modeling

Mice in control groups were fed a normal diet (ND) [LabDiet Picolab Rodent Diet 5053 (minimum protein=20%, crude fat=4.5%, maximum crude fiber=6.0%)] *ad libitum* over the course of the study. Type-1 diabetes is an autoimmune disease that destroys insulin-producing pancreatic β-cells, which we modeled by administering the β-cell toxin STZ (Sigma S0130) to mice at 8 weeks of age fed a ND (ND+STZ; Arora et al., 2009; Gilbert et al., 2011). STZ was prepared in a 7.5 mg/ml sodium citrate buffer (pH 4.5) immediately prior to injection. On five consecutive days, mice were fasted for 4 h, anesthetized with inhaled isoflurane (induction at 2.5-4.5%, maintained at 1-2% evaporated in 1-1.5 liter/min O_2_), before being administered 40 mg/kg intraperitoneal STZ.

Type-2 diabetes is characterized by insulin resistance and the inability of β-cells to upregulate their function. To model this, mice were fed a diabetic diet (DD) (Bio-Serv Mouse Diet F3282; protein=20.5%, crude fat=36%, fiber=0%, carbohydrates=35.7%) *ad libitum* from 4 weeks of age. To model an accelerated version of type-2 diabetes, another group was fed a DD from 4 weeks and administered STZ at 8 weeks of age (DD+STZ).

### MTP-131 administration

In one experiment, mice were injected daily with a 1 mg/kg solution of MTP-131 subcutaneously (provided by Stealth Peptides, Newton, MA, USA) dissolved in 0.9% sterile saline (pH 5.5-6.5) or with saline alone. In another experiment, mice were administered MTP-131 as an ophthalmic-formulated solution (Ocuvia, provided by Stealth Peptides) daily via eye drops [in 0.01 M sodium acetate buffer solution (pH 6.00); 5 μl/eye] or with buffer alone.

### Measures of weight and blood glucose

Mice were weighed, then lightly anesthetized with inhaled isoflurane; a drop of blood was harvested from the tail or submandibular vein. Glucose in the drop was measured with a glucometer (AlphaTRAK II Blood Glucose Monitoring System or One Touch Ultra Mini Blood Glucose Monitoring System). A glucose-tolerance test was also administered periodically. Animals were fasted overnight prior to receiving D-glucose (1.5 g/kg i.p.). Blood glucose was measured as above prior to and after 10, 20, 30, 60 and 120 min D-glucose administration.

### Tests of spatial visual function

Spatial thresholds for opto-kinetic tracking of sine-wave gratings were measured weekly using a virtual optokinetic system (OptoMotry, CerebralMechanics, Medicine Hat, Alberta, Canada) (Prusky et al., 2004). Vertical sine-wave gratings moving at 12°/s or gray of the same mean luminance were projected on four monitors as a virtual cylinder that surrounded an unrestrained mouse standing on a platform at the epicenter. The hub of the cylinder was continually centered between the eyes of the mouse to set the SF of the grating at the mouse's viewing position as it shifted its position. Gray was projected while the mouse was moving, but when movement ceased, the gray was replaced with the grating. Grating rotation under these circumstances elicited reflexive tracking, which was scored via live video using a method of limits procedure with a yes/no criterion. A SF threshold, and contrast thresholds at six spatial frequencies (0.031, 0.064, 0.092, 0.103, 0.192, 0.272 c/d) were generated through each eye separately in a testing session (14 thresholds in ∼30 min). Michelson contrast sensitivity (CS) was calculated using the average screen luminance (maximum−minimum)/(maximum+minimum). Most thresholds were generated under photopic lighting conditions (screen luminance=54 lux), which selectively measures cone-based visual function. Rod-based visual function under scotopic conditions (1 lux) was also assessed in some animals after dark adaptation (>6 h; Alam et al., 2015). For this, 6.3 ND filters (Lee Filters) were placed on the monitors, and infrared lighting and an infrared-sensitive camera (Sony Handycam DCR-HC28, Sony, Japan) were used to image the animal.

### Ophthalmic assessments

A biomicroscope (slit lamp) was used to examine the cornea for clarity, size, surface texture and vascularization; the iris was inspected for pupil size, constriction, reflected luminescence and synechia. The pupil was then dilated with a drop of 0.05% tropicamide ophthalmic solution, and the lens was scored for cataract using a modified version of the Merriam-Focht scoring criterion (Merriam and Focht, 1962; Worgul et al., 1993). An indirect ophthalmoscope was also used to inspect the fundus for damage, degeneration, retinal vessel constriction and optic nerve head abnormalities. A modified MacDonald-Shaddock Scoring System was used to score the optical quality of eyes at 12 and 32 weeks of age.

### Statistical analyses

Using Graphpad Prism 6 software, two-way, repeated-measures ANOVAs were used to make group comparisons. Post-hoc multiple comparisons were performed using the Tukey's or Bonferroni correction methods. Statistical comparisons were considered significantly different at *P*<0.05.
